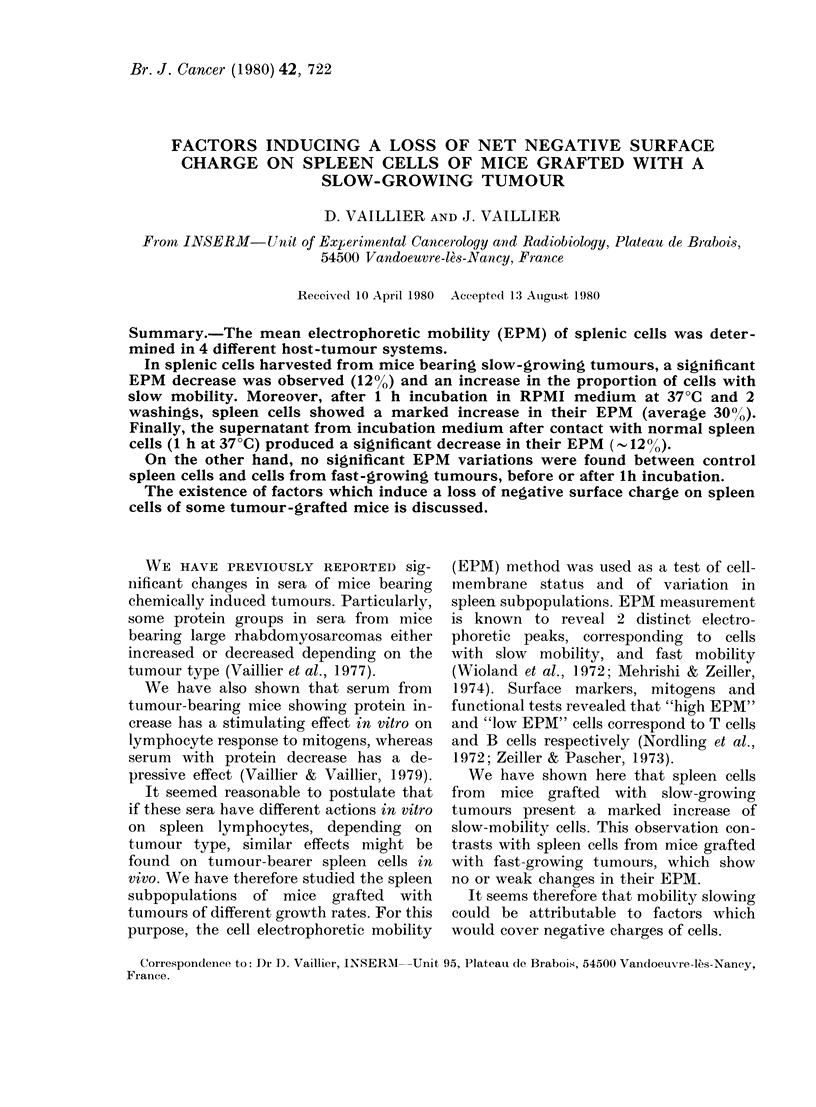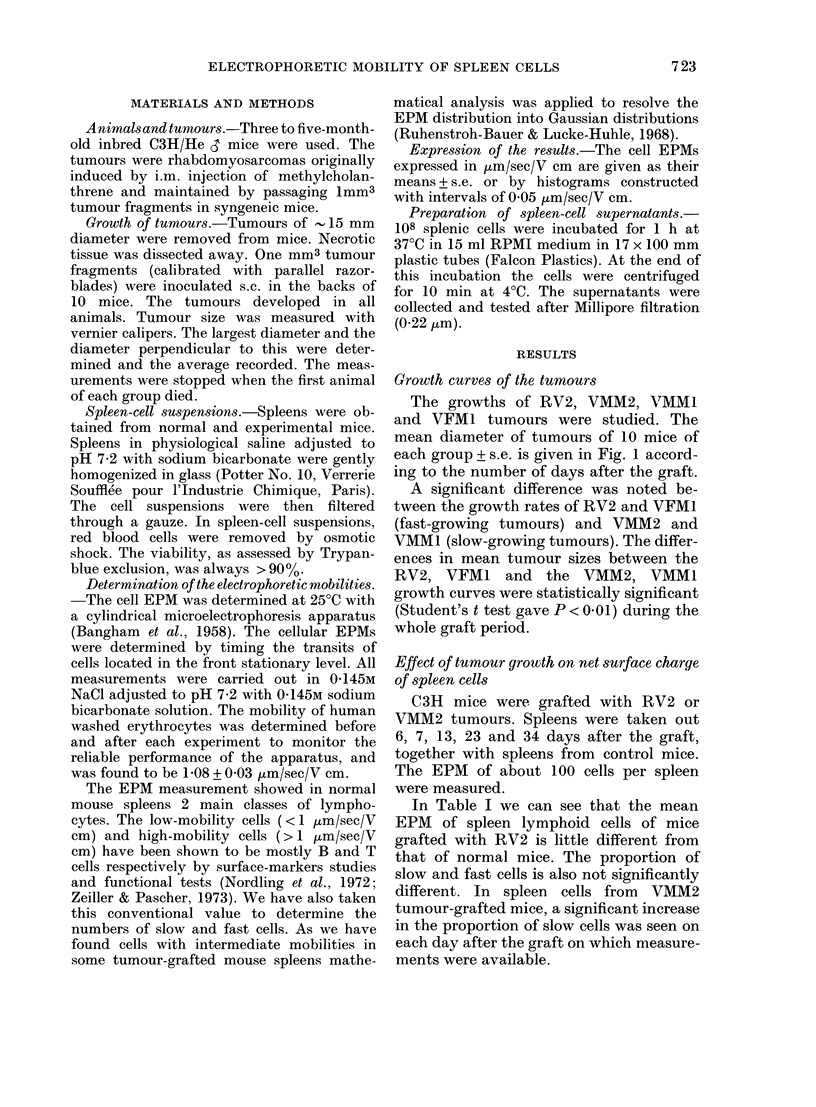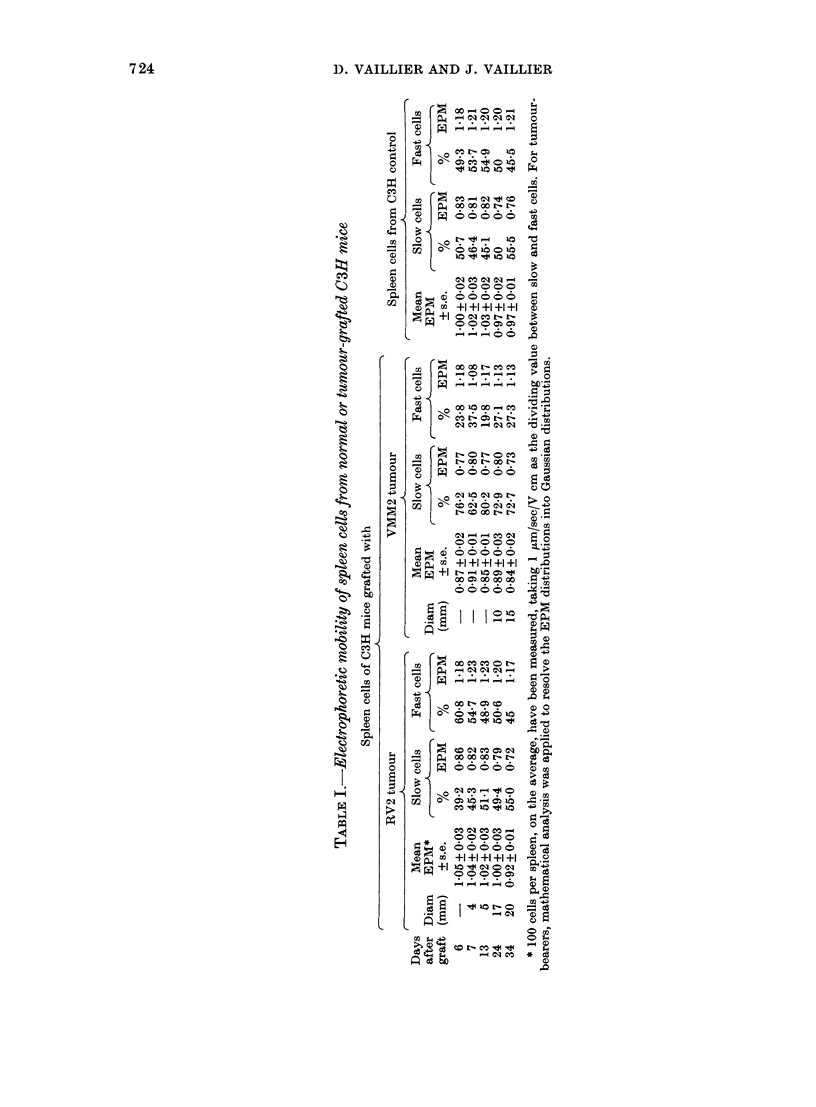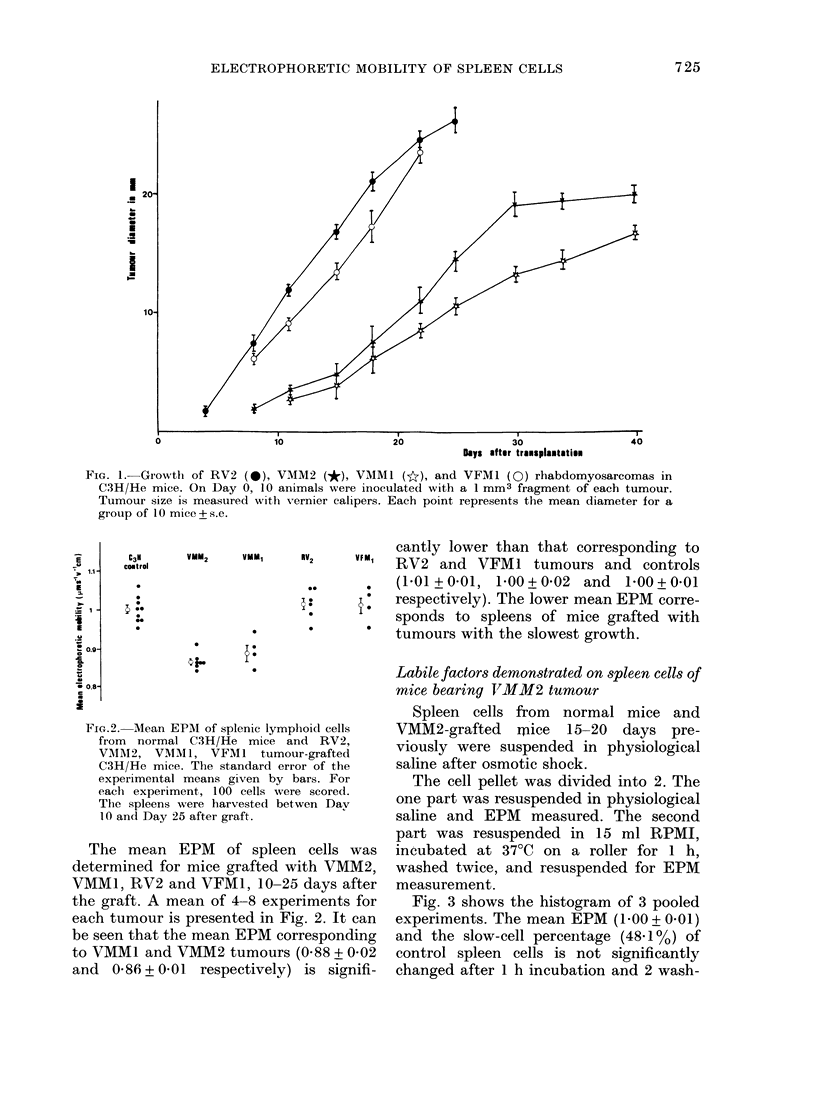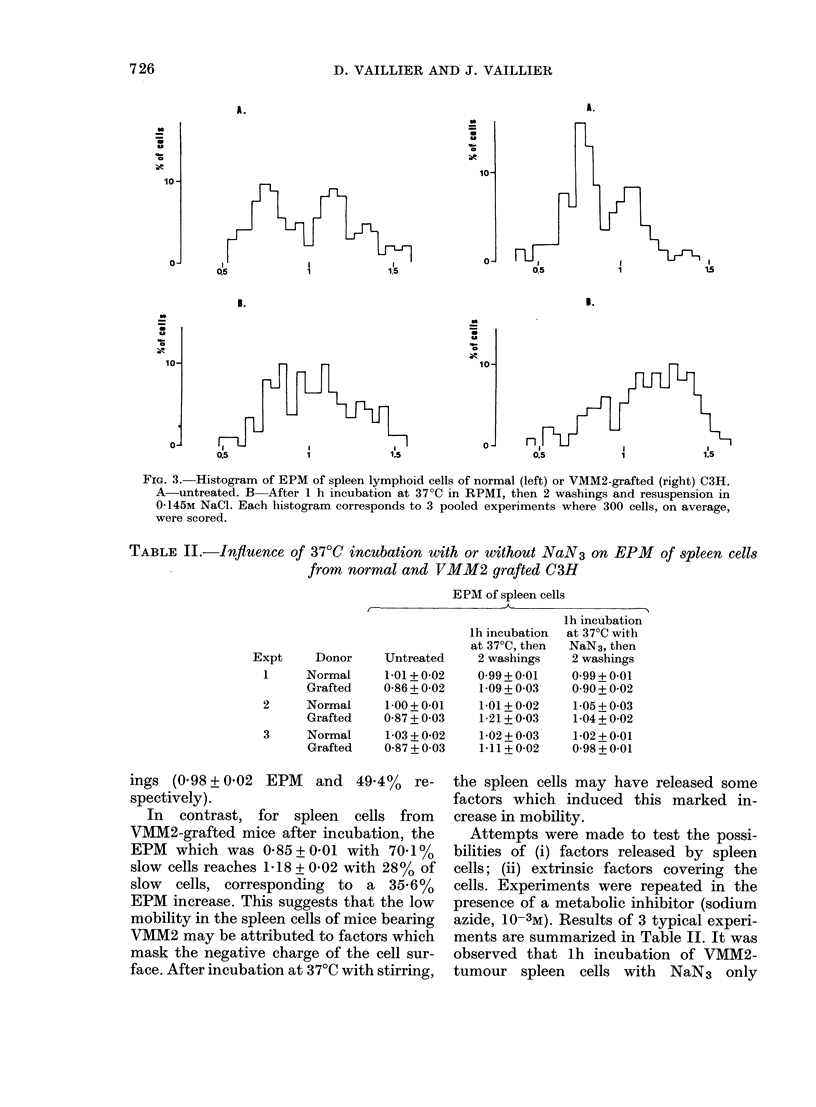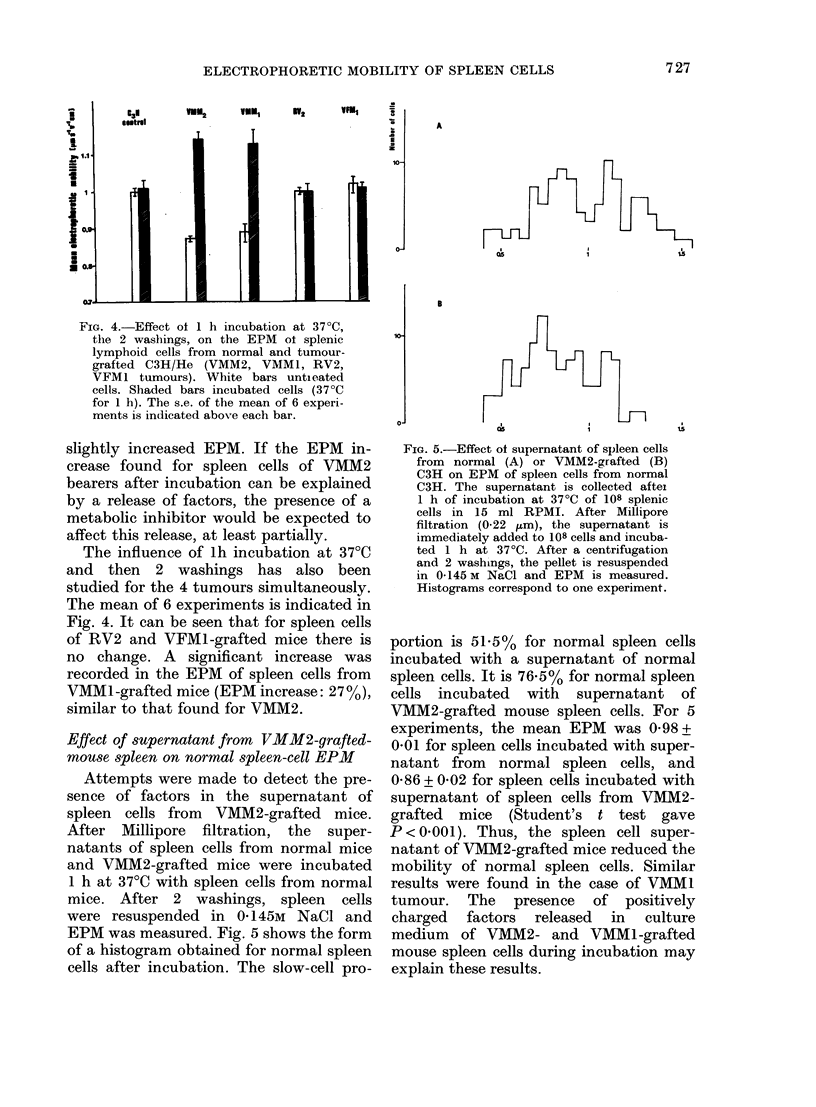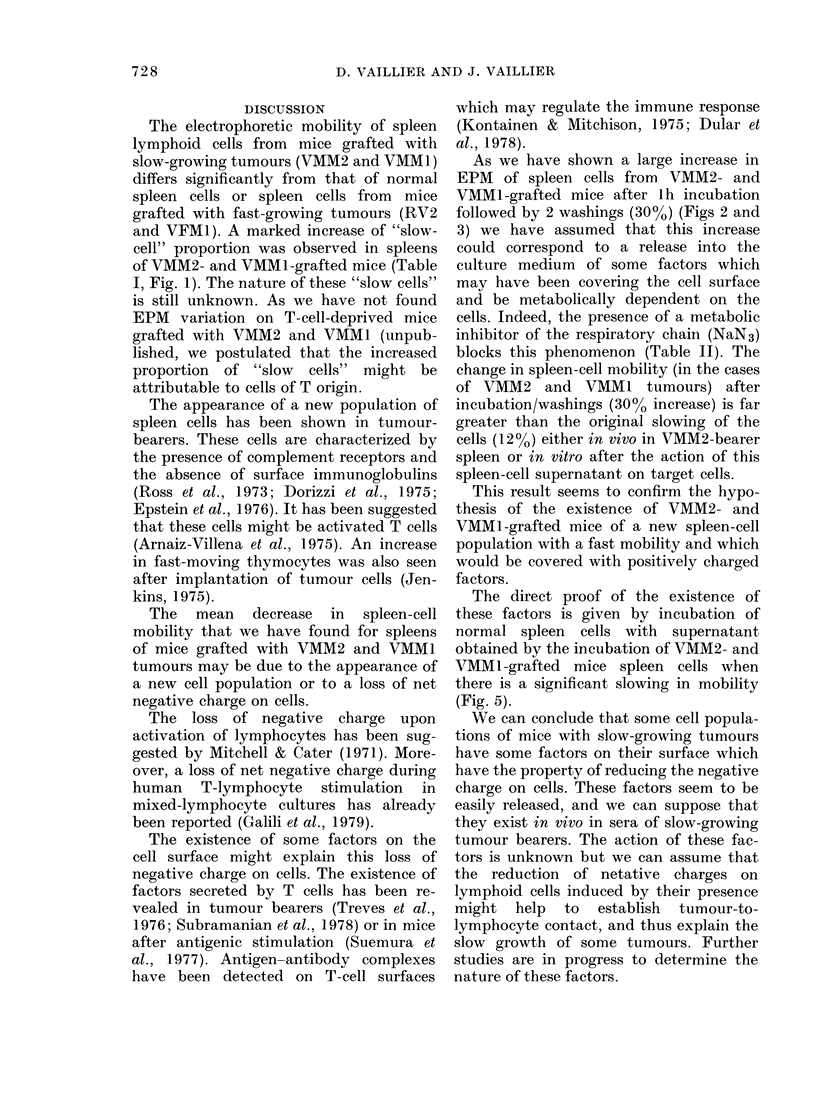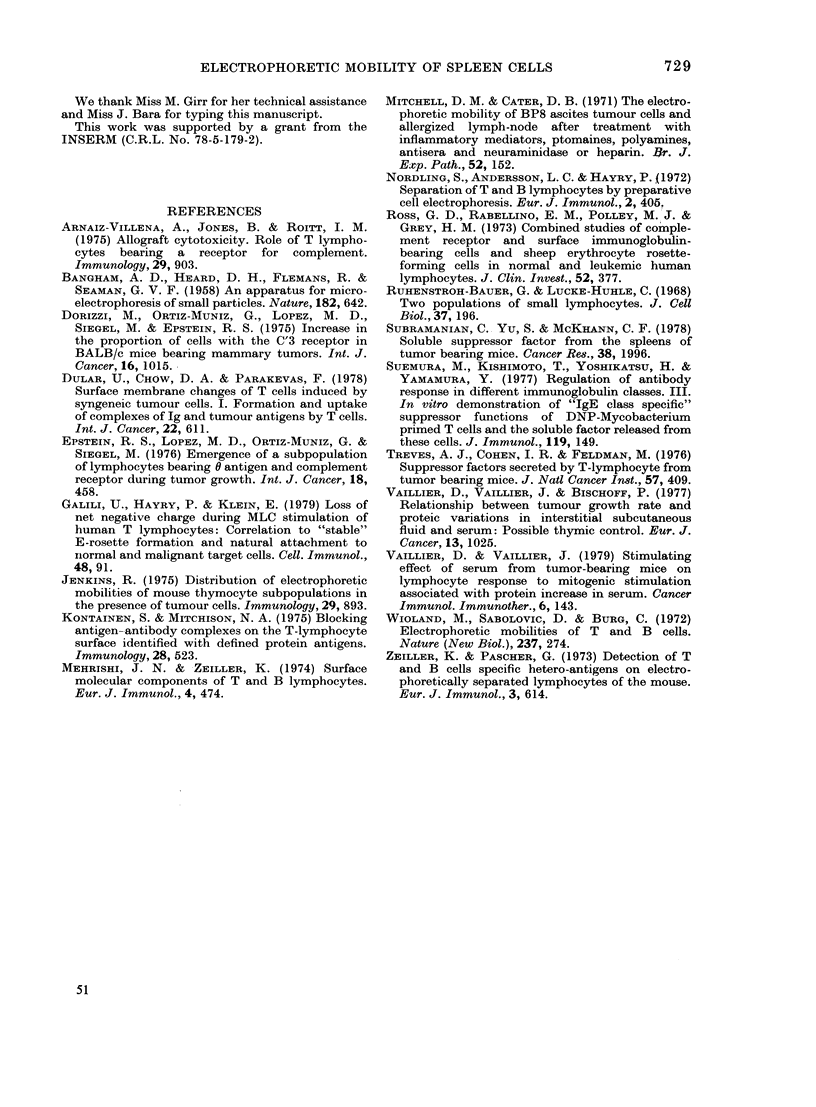# Factors inducing a loss of net negative surface charge on spleen cells of mice grafted with a slow-growing tumour.

**DOI:** 10.1038/bjc.1980.307

**Published:** 1980-11

**Authors:** D. Vaillier, J. Vaillier

## Abstract

The mean electrophoretic mobility (EPM) of splenic cells was determined in 4 different host-tumour systems. In splenic cells harvested from mice bearing slow-growing tumours, a significant EPM decrease was observed (12%) and in increase in the proportion of cells with slow mobility. Moreover, after 1 h incubation in RPMI medium at 37 degrees C and 2 washings, spleen cells showed a marked increase in their EPM (average 30%). Finally, the supernatant from incubation medium after contact with normal spleen cells (1 h at 37 degrees C) produced a significant decrease in their EPM (approximately 12%). On the other hand, no significant EPM variations were found between control spleen cells and cells from fast-growing tumours, before or after 1h incubation. The existence of factors which induce a loss of negative surface charge on spleen cells of some tumour-grafted mice is discussed.


*Br. J. Cancer* (1980) **42**, 722

### FACTORS INDUCING A LOSS OF NET NEGATIVE SURFACE CHARGE ON SPLEEN CELLS OF MICE GRAFTED WITH A SLOW-GROWING TUMOUR

D. VAILLIER AND J. VAILLIER

*From INSERM—Unit of Experimental Cancerology and Radiobiology, Plateau de Brabois, 54500 Vandoeuvre-lès-Nancy, France*

Received 10 April 1980 Accepted 13 August 1980

**Summary.—The mean electrophoretic mobility (EPM) of splenic cells was determined in 4 different host-tumour systems.**

**In splenic cells harvested from mice bearing slow-growing tumours, a significant EPM decrease was observed (12%) and an increase in the proportion of cells with slow mobility. Moreover, after 1 h incubation in RPMI medium at 37°C and 2 washings, spleen cells showed a marked increase in their EPM (average 30%). Finally, the supernatant from incubation medium after contact with normal spleen cells (1 h at 37°C) produced a significant decrease in their EPM (~12%).**

**On the other hand, no significant EPM variations were found between control spleen cells and cells from fast-growing tumours, before or after 1h incubation.**

**The existence of factors which induce a loss of negative surface charge on spleen cells of some tumour-grafted mice is discussed.**

We have previously reported significant changes in sera of mice bearing chemically induced tumours. Particularly, some protein groups in sera from mice bearing large rhabdomyosarcomas either increased or decreased depending on the tumour type (Vaillier *et al.*, 1977).

We have also shown that serum from tumour-bearing mice showing protein increase has a stimulating effect *in vitro* on lymphocyte response to mitogens, whereas serum with protein decrease has a depressive effect (Vaillier & Vaillier, 1979).

It seemed reasonable to postulate that if these sera have different actions *in vitro* on spleen lymphocytes, depending on tumour type, similar effects might be found on tumour-bearer spleen cells *in vivo*. We have therefore studied the spleen subpopulations of mice grafted with tumours of different growth rates. For this purpose, the cell electrophoretic mobility (EPM) method was used as a test of cell-membrane status and of variation in spleen subpopulations. EPM measurement is known to reveal 2 distinct electrophoretic peaks, corresponding to cells with slow mobility, and fast mobility (Wioland *et al.*, 1972; Mehrishi & Zeiller, 1974). Surface markers, mitogens and functional tests revealed that "high EPM" and "low EPM" cells correspond to T cells and B cells respectively (Nordling *et al.*, 1972; Zeiller & Pascher, 1973).

We have shown here that spleen cells from mice grafted with slow-growing tumours present a marked increase of slow-mobility cells. This observation contrasts with spleen cells from mice grafted with fast-growing tumours, which show no or weak changes in their EPM.

It seems therefore that mobility slowing could be attributable to factors which would cover negative charges of cells.

Correspondence to: Dr D. Vaillier, INSERM—Unit 95, Plateau de Brabois, 54500 Vandoeuvre-lès-Nancy, France.

### MATERIALS AND METHODS

*Animals and tumours.*—Three to five-month-old inbred C3H/He ♂ mice were used. The tumours were rhabdomyosarcomas originally induced by i.m. injection of methylcholanthrene and maintained by passaging 1mm$^3$ tumour fragments in syngeneic mice.

*Growth of tumours.*—Tumours of ~15 mm diameter were removed from mice. Necrotic tissue was dissected away. One mm$^3$ tumour fragments (calibrated with parallel razor-blades) were inoculated s.c. in the backs of 10 mice. The tumours developed in all animals. Tumour size was measured with vernier calipers. The largest diameter and the diameter perpendicular to this were determined and the average recorded. The measurements were stopped when the first animal of each group died.

*Spleen-cell suspensions.*—Spleens were obtained from normal and experimental mice. Spleens in physiological saline adjusted to pH 7·2 with sodium bicarbonate were gently homogenized in glass (Potter No. 10, Verrerie Soufflée pour l'Industrie Chimique, Paris). The cell suspensions were then filtered through a gauze. In spleen-cell suspensions, red blood cells were removed by osmotic shock. The viability, as assessed by Trypan-blue exclusion, was always >90%.

*Determination of the electrophoretic mobilities.*—The cell EPM was determined at 25°C with a cylindrical microelectrophoresis apparatus (Bangham *et al.*, 1958). The cellular EPMs were determined by timing the transits of cells located in the front stationary level. All measurements were carried out in 0·145M NaCl adjusted to pH 7·2 with 0·145M sodium bicarbonate solution. The mobility of human washed erythrocytes was determined before and after each experiment to monitor the reliable performance of the apparatus, and was found to be 1·08 ± 0·03 μm/sec/V cm.

The EPM measurement showed in normal mouse spleens 2 main classes of lymphocytes. The low-mobility cells (<1 μm/sec/V cm) and high-mobility cells (>1 μm/sec/V cm) have been shown to be mostly B and T cells respectively by surface-markers studies and functional tests (Nordling *et al.*, 1972; Zeiller & Pascher, 1973). We have also taken this conventional value to determine the numbers of slow and fast cells. As we have found cells with intermediate mobilities in some tumour-grafted mouse spleens mathematical analysis was applied to resolve the EPM distribution into Gaussian distributions (Ruhenstroh-Bauer & Lucke-Huhle, 1968).

*Expression of the results.*—The cell EPMs expressed in μm/sec/V cm are given as their means ± s.e. or by histograms constructed with intervals of 0·05 μm/sec/V cm.

*Preparation of spleen-cell supernatants.*—10$^8$ splenic cells were incubated for 1 h at 37°C in 15 ml RPMI medium in 17 × 100 mm plastic tubes (Falcon Plastics). At the end of this incubation the cells were centrifuged for 10 min at 4°C. The supernatants were collected and tested after Millipore filtration (0·22 μm).

### RESULTS

### *Growth curves of the tumours*

The growths of RV2, VMM2, VMM1 and VFM1 tumours were studied. The mean diameter of tumours of 10 mice of each group ± s.e. is given in Fig. 1 according to the number of days after the graft.

A significant difference was noted between the growth rates of RV2 and VFM1 (fast-growing tumours) and VMM2 and VMM1 (slow-growing tumours). The differences in mean tumour sizes between the RV2, VFM1 and the VMM2, VMM1 growth curves were statistically significant (Student's $t$ test gave $P < 0{\cdot}01$) during the whole graft period.

### *Effect of tumour growth on net surface charge of spleen cells*

C3H mice were grafted with RV2 or VMM2 tumours. Spleens were taken out 6, 7, 13, 23 and 34 days after the graft, together with spleens from control mice. The EPM of about 100 cells per spleen were measured.

In Table I we can see that the mean EPM of spleen lymphoid cells of mice grafted with RV2 is little different from that of normal mice. The proportion of slow and fast cells is also not significantly different. In spleen cells from VMM2 tumour-grafted mice, a significant increase in the proportion of slow cells was seen on each day after the graft on which measurements were available.

TABLE I.—*Electrophoretic mobility of spleen cells from normal or tumour-grafted C3H mice*

| | Spleen cells of C3H mice grafted with | | | | | | | | | | | | | Spleen cells from C3H control | | | | |
|---|---|---|---|---|---|---|---|---|---|---|---|---|---|---|---|---|---|---|
| | RV2 tumour | | | | | | VMM2 tumour | | | | | | | | | | | |
| Days after graft | Diam (mm) | Mean EPM* ±s.e. | Slow cells % | Slow cells EPM | Fast cells % | Fast cells EPM | Diam (mm) | Mean EPM ±s.e. | Slow cells % | Slow cells EPM | Fast cells % | Fast cells EPM | Mean EPM ±s.e. | Slow cells % | Slow cells EPM | Fast cells % | Fast cells EPM | |
| 6 | — | 1·05±0·03 | 39·2 | 0·86 | 60·8 | 1·18 | — | 0·87±0·02 | 76·2 | 0·77 | 23·8 | 1·18 | 1·00±0·02 | 50·7 | 0·83 | 49·3 | 1·18 | |
| 7 | 4 | 1·04±0·02 | 45·3 | 0·82 | 54·7 | 1·23 | — | 0·91±0·01 | 62·5 | 0·80 | 37·5 | 1·08 | 1·02±0·03 | 46·4 | 0·81 | 53·7 | 1·21 | |
| 13 | 5 | 1·02±0·03 | 51·1 | 0·83 | 48·9 | 1·23 | — | 0·85±0·01 | 80·2 | 0·77 | 19·8 | 1·17 | 1·03±0·02 | 45·1 | 0·82 | 54·9 | 1·20 | |
| 24 | 17 | 1·00±0·03 | 49·4 | 0·79 | 50·6 | 1·20 | 10 | 0·89±0·03 | 72·9 | 0·80 | 27·1 | 1·13 | 0·97±0·02 | 50 | 0·74 | 50 | 1·20 | |
| 34 | 20 | 0·92±0·01 | 55·0 | 0·72 | 45 | 1·17 | 15 | 0·84±0·02 | 72·7 | 0·73 | 27·3 | 1·13 | 0·97±0·01 | 55·5 | 0·76 | 45·5 | 1·21 | |

* 100 cells per spleen, on the average, have been measured, taking 1 μm/sec/V cm as the dividing value between slow and fast cells. For tumour-bearers, mathematical analysis was applied to resolve the EPM distributions into Gaussian distributions.

FIG. 1.—Growth of RV2 (●), VMM2 (★), VMM1 (☆), and VFM1 (○) rhabdomyosarcomas in C3H/He mice. On Day 0, 10 animals were inoculated with a 1 mm$^3$ fragment of each tumour. Tumour size is measured with vernier calipers. Each point represents the mean diameter for a group of 10 mice ± s.e.

FIG. 2.—Mean EPM of splenic lymphoid cells from normal C3H/He mice and RV2, VMM2, VMM1, VFM1 tumour-grafted C3H/He mice. The standard error of the experimental means given by bars. For each experiment, 100 cells were scored. The spleens were harvested betwen Day 10 and Day 25 after graft.

The mean EPM of spleen cells was determined for mice grafted with VMM2, VMM1, RV2 and VFM1, 10–25 days after the graft. A mean of 4–8 experiments for each tumour is presented in Fig. 2. It can be seen that the mean EPM corresponding to VMM1 and VMM2 tumours (0·88 ± 0·02 and 0·86 ± 0·01 respectively) is significantly lower than that corresponding to RV2 and VFM1 tumours and controls (1·01 ± 0·01, 1·00 ± 0·02 and 1·00 ± 0·01 respectively). The lower mean EPM corresponds to spleens of mice grafted with tumours with the slowest growth.

*Labile factors demonstrated on spleen cells of mice bearing VMM2 tumour*

Spleen cells from normal mice and VMM2-grafted mice 15–20 days previously were suspended in physiological saline after osmotic shock.

The cell pellet was divided into 2. The one part was resuspended in physiological saline and EPM measured. The second part was resuspended in 15 ml RPMI, incubated at 37°C on a roller for 1 h, washed twice, and resuspended for EPM measurement.

Fig. 3 shows the histogram of 3 pooled experiments. The mean EPM (1·00 ± 0·01) and the slow-cell percentage (48·1%) of control spleen cells is not significantly changed after 1 h incubation and 2 wash-

FIG. 3.—Histogram of EPM of spleen lymphoid cells of normal (left) or VMM2-grafted (right) C3H. A—untreated. B—After 1 h incubation at 37°C in RPMI, then 2 washings and resuspension in 0·145M NaCl. Each histogram corresponds to 3 pooled experiments where 300 cells, on average, were scored.

TABLE II.—*Influence of 37°C incubation with or without $NaN_3$ on EPM of spleen cells from normal and VMM2 grafted C3H*

| | | EPM of spleen cells | | |
|---|---|---|---|---|
| Expt | Donor | Untreated | 1h incubation at 37°C, then 2 washings | 1h incubation at 37°C with $NaN_3$, then 2 washings |
| 1 | Normal | 1·01 ± 0·02 | 0·99 ± 0·01 | 0·99 ± 0·01 |
| | Grafted | 0·86 ± 0·02 | 1·09 ± 0·03 | 0·90 ± 0·02 |
| 2 | Normal | 1·00 ± 0·01 | 1·01 ± 0·02 | 1·05 ± 0·03 |
| | Grafted | 0·87 ± 0·03 | 1·21 ± 0·03 | 1·04 ± 0·02 |
| 3 | Normal | 1·03 ± 0·02 | 1·02 ± 0·03 | 1·02 ± 0·01 |
| | Grafted | 0·87 ± 0·03 | 1·11 ± 0·02 | 0·98 ± 0·01 |

ings (0·98 ± 0·02 EPM and 49·4% respectively).

In contrast, for spleen cells from VMM2-grafted mice after incubation, the EPM which was 0·85 ± 0·01 with 70·1% slow cells reaches 1·18 ± 0·02 with 28% of slow cells, corresponding to a 35·6% EPM increase. This suggests that the low mobility in the spleen cells of mice bearing VMM2 may be attributed to factors which mask the negative charge of the cell surface. After incubation at 37°C with stirring, the spleen cells may have released some factors which induced this marked increase in mobility.

Attempts were made to test the possibilities of (i) factors released by spleen cells; (ii) extrinsic factors covering the cells. Experiments were repeated in the presence of a metabolic inhibitor (sodium azide, $10^{-3}$M). Results of 3 typical experiments are summarized in Table II. It was observed that 1h incubation of VMM2-tumour spleen cells with $NaN_3$ only

FIG. 4.—Effect of 1 h incubation at 37°C, the 2 washings, on the EPM of splenic lymphoid cells from normal and tumour-grafted C3H/He (VMM2, VMM1, RV2, VFM1 tumours). White bars untreated cells. Shaded bars incubated cells (37°C for 1 h). The s.e. of the mean of 6 experiments is indicated above each bar.

FIG. 5.—Effect of supernatant of spleen cells from normal (A) or VMM2-grafted (B) C3H on EPM of spleen cells from normal C3H. The supernatant is collected after 1 h of incubation at 37°C of $10^8$ splenic cells in 15 ml RPMI. After Millipore filtration (0·22 μm), the supernatant is immediately added to $10^8$ cells and incubated 1 h at 37°C. After a centrifugation and 2 washings, the pellet is resuspended in 0·145 M NaCl and EPM is measured. Histograms correspond to one experiment.

slightly increased EPM. If the EPM increase found for spleen cells of VMM2 bearers after incubation can be explained by a release of factors, the presence of a metabolic inhibitor would be expected to affect this release, at least partially.

The influence of 1h incubation at 37°C and then 2 washings has also been studied for the 4 tumours simultaneously. The mean of 6 experiments is indicated in Fig. 4. It can be seen that for spleen cells of RV2 and VFM1-grafted mice there is no change. A significant increase was recorded in the EPM of spleen cells from VMM1-grafted mice (EPM increase: 27%), similar to that found for VMM2.

### *Effect of supernatant from VMM2-grafted-mouse spleen on normal spleen-cell EPM*

Attempts were made to detect the presence of factors in the supernatant of spleen cells from VMM2-grafted mice. After Millipore filtration, the supernatants of spleen cells from normal mice and VMM2-grafted mice were incubated 1 h at 37°C with spleen cells from normal mice. After 2 washings, spleen cells were resuspended in 0·145M NaCl and EPM was measured. Fig. 5 shows the form of a histogram obtained for normal spleen cells after incubation. The slow-cell proportion is 51·5% for normal spleen cells incubated with a supernatant of normal spleen cells. It is 76·5% for normal spleen cells incubated with supernatant of VMM2-grafted mouse spleen cells. For 5 experiments, the mean EPM was $0{\cdot}98 \pm 0{\cdot}01$ for spleen cells incubated with supernatant from normal spleen cells, and $0{\cdot}86 \pm 0{\cdot}02$ for spleen cells incubated with supernatant of spleen cells from VMM2-grafted mice (Student's $t$ test gave $P < 0{\cdot}001$). Thus, the spleen cell supernatant of VMM2-grafted mice reduced the mobility of normal spleen cells. Similar results were found in the case of VMM1 tumour. The presence of positively charged factors released in culture medium of VMM2- and VMM1-grafted mouse spleen cells during incubation may explain these results.

### DISCUSSION

The electrophoretic mobility of spleen lymphoid cells from mice grafted with slow-growing tumours (VMM2 and VMM1) differs significantly from that of normal spleen cells or spleen cells from mice grafted with fast-growing tumours (RV2 and VFM1). A marked increase of "slow-cell" proportion was observed in spleens of VMM2- and VMM1-grafted mice (Table I, Fig. 1). The nature of these "slow cells" is still unknown. As we have not found EPM variation on T-cell-deprived mice grafted with VMM2 and VMM1 (unpublished, we postulated that the increased proportion of "slow cells" might be attributable to cells of T origin.

The appearance of a new population of spleen cells has been shown in tumour-bearers. These cells are characterized by the presence of complement receptors and the absence of surface immunoglobulins (Ross *et al.*, 1973; Dorizzi *et al.*, 1975; Epstein *et al.*, 1976). It has been suggested that these cells might be activated T cells (Arnaiz-Villena *et al.*, 1975). An increase in fast-moving thymocytes was also seen after implantation of tumour cells (Jenkins, 1975).

The mean decrease in spleen-cell mobility that we have found for spleens of mice grafted with VMM2 and VMM1 tumours may be due to the appearance of a new cell population or to a loss of net negative charge on cells.

The loss of negative charge upon activation of lymphocytes has been suggested by Mitchell & Cater (1971). Moreover, a loss of net negative charge during human T-lymphocyte stimulation in mixed-lymphocyte cultures has already been reported (Galili *et al.*, 1979).

The existence of some factors on the cell surface might explain this loss of negative charge on cells. The existence of factors secreted by T cells has been revealed in tumour bearers (Treves *et al.*, 1976; Subramanian *et al.*, 1978) or in mice after antigenic stimulation (Suemura *et al.*, 1977). Antigen–antibody complexes have been detected on T-cell surfaces which may regulate the immune response (Kontainen & Mitchison, 1975; Dular *et al.*, 1978).

As we have shown a large increase in EPM of spleen cells from VMM2- and VMM1-grafted mice after 1h incubation followed by 2 washings (30%) (Figs 2 and 3) we have assumed that this increase could correspond to a release into the culture medium of some factors which may have been covering the cell surface and be metabolically dependent on the cells. Indeed, the presence of a metabolic inhibitor of the respiratory chain ($NaN_3$) blocks this phenomenon (Table II). The change in spleen-cell mobility (in the cases of VMM2 and VMM1 tumours) after incubation/washings (30% increase) is far greater than the original slowing of the cells (12%) either *in vivo* in VMM2-bearer spleen or *in vitro* after the action of this spleen-cell supernatant on target cells.

This result seems to confirm the hypothesis of the existence of VMM2- and VMM1-grafted mice of a new spleen-cell population with a fast mobility and which would be covered with positively charged factors.

The direct proof of the existence of these factors is given by incubation of normal spleen cells with supernatant obtained by the incubation of VMM2- and VMM1-grafted mice spleen cells when there is a significant slowing in mobility (Fig. 5).

We can conclude that some cell populations of mice with slow-growing tumours have some factors on their surface which have the property of reducing the negative charge on cells. These factors seem to be easily released, and we can suppose that they exist *in vivo* in sera of slow-growing tumour bearers. The action of these factors is unknown but we can assume that the reduction of netative charges on lymphoid cells induced by their presence might help to establish tumour-to-lymphocyte contact, and thus explain the slow growth of some tumours. Further studies are in progress to determine the nature of these factors.

We thank Miss M. Girr for her technical assistance and Miss J. Bara for typing this manuscript.

This work was supported by a grant from the INSERM (C.R.L. No. 78-5-179-2).

### REFERENCES

ARNAIZ-VILLENA, A., JONES, B. & ROITT, I. M. (1975) Allograft cytotoxicity. Role of T lymphocytes bearing a receptor for complement. *Immunology*, **29**, 903.

BANGHAM, A. D., HEARD, D. H., FLEMANS, R. & SEAMAN, G. V. F. (1958) An apparatus for microelectrophoresis of small particles. *Nature*, **182**, 642.

DORIZZI, M., ORTIZ-MUNIZ, G., LOPEZ, M. D., SIEGEL, M. & EPSTEIN, R. S. (1975) Increase in the proportion of cells with the C′3 receptor in BALB/c mice bearing mammary tumors. *Int. J. Cancer*, **16**, 1015.

DULAR, U., CHOW, D. A. & PARAKEVAS, F. (1978) Surface membrane changes of T cells induced by syngeneic tumour cells. I. Formation and uptake of complexes of Ig and tumour antigens by T cells. *Int. J. Cancer*, **22**, 611.

EPSTEIN, R. S., LOPEZ, M. D., ORTIZ-MUNIZ, G. & SIEGEL, M. (1976) Emergence of a subpopulation of lymphocytes bearing $\theta$ antigen and complement receptor during tumor growth. *Int. J. Cancer*, **18**, 458.

GALILI, U., HAYRY, P. & KLEIN, E. (1979) Loss of net negative charge during MLC stimulation of human T lymphocytes: Correlation to "stable" E-rosette formation and natural attachment to normal and malignant target cells. *Cell. Immunol.*, **48**, 91.

JENKINS, R. (1975) Distribution of electrophoretic mobilities of mouse thymocyte subpopulations in the presence of tumour cells. *Immunology*, **29**, 893.

KONTAINEN, S. & MITCHISON, N. A. (1975) Blocking antigen-antibody complexes on the T-lymphocyte surface identified with defined protein antigens. *Immunology*, **28**, 523.

MEHRISHI, J. N. & ZEILLER, K. (1974) Surface molecular components of T and B lymphocytes. *Eur. J. Immunol.*, **4**, 474.

MITCHELL, D. M. & CATER, D. B. (1971) The electrophoretic mobility of BP8 ascites tumour cells and allergized lymph-node after treatment with inflammatory mediators, ptomaines, polyamines, antisera and neuraminidase or heparin. *Br. J. Exp. Path.*, **52**, 152.

NORDLING, S., ANDERSSON, L. C. & HAYRY, P. (1972) Separation of T and B lymphocytes by preparative cell electrophoresis. *Eur. J. Immunol.*, **2**, 405.

ROSS, G. D., RABELLINO, E. M., POLLEY, M. J. & GREY, H. M. (1973) Combined studies of complement receptor and surface immunoglobulin-bearing cells and sheep erythrocyte rosette-forming cells in normal and leukemic human lymphocytes. *J. Clin. Invest.*, **52**, 377.

RUHENSTROH-BAUER, G. & LUCKE-HUHLE, C. (1968) Two populations of small lymphocytes. *J. Cell Biol.*, **37**, 196.

SUBRAMANIAN, C. YU, S. & MCKHANN, C. F. (1978) Soluble suppressor factor from the spleens of tumor bearing mice. *Cancer Res.*, **38**, 1996.

SUEMURA, M., KISHIMOTO, T., YOSHIKATSU, H. & YAMAMURA, Y. (1977) Regulation of antibody response in different immunoglobulin classes. III. *In vitro* demonstration of "IgE class specific" suppressor functions of DNP-Mycobacterium primed T cells and the soluble factor released from these cells. *J. Immunol.*, **119**, 149.

TREVES, A. J., COHEN, I. R. & FELDMAN, M. (1976) Suppressor factors secreted by T-lymphocyte from tumor bearing mice. *J. Natl Cancer Inst.*, **57**, 409.

VAILLIER, D., VAILLIER, J. & BISCHOFF, P. (1977) Relationship between tumour growth rate and proteic variations in interstitial subcutaneous fluid and serum: Possible thymic control. *Eur. J. Cancer*, **13**, 1025.

VAILLIER, D. & VAILLIER, J. (1979) Stimulating effect of serum from tumor-bearing mice on lymphocyte response to mitogenic stimulation associated with protein increase in serum. *Cancer Immunol. Immunother.*, **6**, 143.

WIOLAND, M., SABOLOVIC, D. & BURG, C. (1972) Electrophoretic mobilities of T and B cells. *Nature* (*New Biol.*), **237**, 274.

ZEILLER, K. & PASCHER, G. (1973) Detection of T and B cells specific hetero-antigens on electrophoretically separated lymphocytes of the mouse. *Eur. J. Immunol.*, **3**, 614.